# Maternal Quality of Life During the Transition to Motherhood

**DOI:** 10.5812/ircmj.8443

**Published:** 2014-05-05

**Authors:** Forough Mortazavi, Seyed Abbas Mousavi, Reza Chaman, Ahmad Khosravi

**Affiliations:** 1 School of Nursing and Midwifery, Sabzevar University of Medical Sciences, Sabzevar, IR Iran; 2 School of Medicine, Golestan University of Medical Sciences, Gorgan, IR Iran; 3 Department of Community Medicine, School of Medicine, Yasuj University of Medical Sciences, Yasuj, IR Iran; 4 Center for Health Related Social and Behavioral Sciences Research, Shahroud University of Medical Sciences, Shahroud, IR Iran

**Keywords:** Quality of Life, Postpartum Period, Pregnancy, Women

## Abstract

**Background:**

One of the elements of the broadening focus of health care beyond its traditional and restricted concept has been the increasing adoption quality of life approach to health care research and practice.

**Objectives:**

To investigate the QOL of women in the third trimester of pregnancy and at 8 weeks postpartum and factors associated with the overall QOL.

**Materials and Methods:**

Three hundred and fifty seven pregnant women attending urban health centers in Shahroud city, located in Northeast of Iran, completed the World Health Organization Quality of Life questionnaire (WHOQOL)-BREF and General Health Questionnaire (GHQ28) in the third trimester of pregnancy and at 8 weeks postpartum. Also, breastfeeding difficulties were assessed at 4 weeks postpartum. Statistical analysis was performed using SPSS 18 for descriptive statistics, paired t-test, linear regression and multiple regression analysis.

**Results:**

There were significant differences between the ante-and postnatal periods in mean scores in the physical (P < 0.001) and social relationship (P = 0.033) aspects of QOL. Multiple regression analysis revealed that factor adversely affected the global score of the QOL in the antenatal period was antepartum psychological disorders. Factors that adversely affected the global score of QOL in the postnatal period were postpartum psychological disorders, breastfeeding difficulties, multiparity, higher pregnancy weight gain, and cesarean.

**Conclusions:**

Results indicated that in this low risk group of women physical health and social relationship improved from pregnancy to postpartum. Interventions to promote psychological status during pregnancy and early postpartum should be designed.

## 1. Background

In recent decades, the traditional narrow concept of health has been replaced with a broad, holistic and positive concept of health epitomized by the WHO definition as “not merely the absence of disease or infirmity” but “a state of complete physical, mental and social well-being” (1). In the field of maternity care, decreasing morbidity and mortality rates in recent decades have prepared the ground for other expectations like enhancing the quality of life (QOL), and the focus of antenatal and postnatal care in developed countries has expanded from its traditional goal of preventing, detecting and managing problems and complications (2). It now includes broader aims such as “supporting psychological adaptation to pregnancy”. This approach reflects the increasing shift of emphasis to the QOL in healthcare research and practice (3).

Pregnancy, childbirth and facing newborn baby’s needs in the early postpartum, are common events in the life of most women, which influence all aspects of their lives (4). Some studies have reported that compared to pre-pregnancy conditions, physical performance of women and their perception of their level of health and well-being decrease during pregnancy (5, 6). Although most of the physical changes during pregnancy reverse after birth and the body returns to its normal state within 8 weeks of postpartum, women may experience many physical and mental symptoms relating to childbirth during this critical period (7). Results of a study indicated that one or more health problems such as tiredness, backache, sexual problems, hemorrhoids, perineal pain and depression were reported by 94% of the women in the first six postnatal months (8). It is also evident that the experience of pain and fatigue can negatively affect QOL after birth (9). Also, during the early postpartum period, women have to deal with various problems relating feeding the baby (4). A Pakistani study found that difficulty in breastfeeding at birth was significantly associated with postpartum anxiety and depression (10). Results of another study demonstrated a relationship between maternal emotional well-being and physical health in the postnatal period (11).

However, despite the importance of this period of transition, there are only a limited number studies on the QOL of women in pregnancy or postpartum considering the important events such as mode of delivery, breastfeeding difficulties and psychological disorders during these periods. Also, there are few studies examining the QOL of women in both pregnancy and postpartum to clarify how it changes during this period. Two studies have reported that compared to pre-pregnancy conditions, physical performance of women and their perception of their level of health and well-being decrease during pregnancy or postpartum (7, 8). The findings of another study, which examined the effects of pregnancy complications on women’s QOL, reported that women with preterm birth or hypertensive disorders had significantly lower QOL scores on the physical domain during pregnancy than those without complications (10). Contrary to most studies which reported lower levels of physical and mental health during pregnancy and specially early postpartum, Mota N et al. reported that pregnant women had a lower likelihood of mental disorder than both non-pregnant and past year pregnant women (11).

Considering the WHO’s emphasis on abandoning a merely “mechanistic model of medicine” (1) and its initiative in developing WHOQOL-100 and WHOQOL-BREF instruments (12), it is surprising to find only a small number studies on the QOL during pregnancy or postpartum using this instruments that cover specific health areas.

## 2. Objectives

With regard to the cultural differences and expectations of women, the aims of this study is therefore to investigate the QOL of women in the third trimester of pregnancy and early postpartum and also the factors associated with QOL during this period using WHOQOL-BREF instrument.

## 3. Materials and Methods

This study was started in May 2011 in urban health centers in Shahroud city, which is located in Northeast of Iran. Ethics Committee of the Shahroud University of Medical Sciences (approval No. 900.02) approved the study protocol. We calculated the sample size at 343 using the following formula: [n = 2(Z _1-α/2_ + Z _1-β_)^2^ × σ^2^ ÷ δ^2^]. In the above formula, σ (standard deviation of QOL) was set at 14 based on the results of a previous study (13). Also, α (type one error), β (type two error), and δ (expected effect size) were set at 5%, 20%, and 3 respectively. We increased the sample size to cover for the possible loss of participants in the follow up observations. Finally, 357 of the 390 women who attended Shahroud Health Centers to receive prenatal care and met the inclusion criteria accepted to participate in the study and gave informed consent of which 340 were followed up until 8 weeks postpartum. They were selected over 6 months using non-probability sampling method. The inclusion criteria was gestational age more than 28 weeks and absence of major psychological and medical problems (e.g. depression, disabilities, and drug intake) and the exclusion criteria were fetal death, infant abnormality, infant death during the first 8 weeks of postpartum, and acute stressful events during the course of study (e.g. loss of a family member or divorce). After explaining the aims of study and obtaining written informed consent from women, women were given instructions on how to fill out the questionnaires. The participants completed the WHOQOL-BREF and GHQ28 in the third trimester of pregnancy and at 8 weeks postpartum. They completed the breastfeeding experience scale at 4 weeks postpartum. Midwives of health centers were responsible of distributing and gathering questionnaires.

### 3.1. Instruments

#### 3.1.1. Interview Form

An interview form containing personal information (i.e., age, years of education, occupation, family income, housing, preconception health) and obstetrical information (parity, wantedness of pregnancy, mode of delivery, BMI, weight gain in pregnancy, women hospitalization during pregnancy and postpartum, infant hospitalization, breastfeeding method, pregnancy complications) were completed during the third trimester of pregnancy and at the first visit postpartum. Information about BMI, and weight gain during pregnancy was collected by the midwives in the health centers and was routinely entered in women’s files.

#### 3.1.2. WHOQOL-BREF

World Health Organization developed the WHOQOL-BREF as a shortened version of the WHOQOL-100 instrument (12). It contains 24 questions in four domains: physical, psychological, social relationships and environment. There are also 2 more questions that are examined separately: question 1 asks about an individual’s overall perception of her QOL and question 2 asks about an individual’s overall perception of her health. The items are rated on a 5-point Likert scale. The raw domain scores can be transformed to a 0-100 scale. Each domain requires that a minimum number of questions be answered in order to generate a score. We followed the WHOQOL- BREF scoring guideline to score missing data in the questionnaire. Where an item was missed, we substituted the mean of other items in the domain. Also, where more than two items were missed from a domain, we did not calculate the domain score.

A previous study investigated the validity and reliability of Farsi version of WHOQOL-BREF in a sample of Tehrani adults and indicated that all its domains met the minimum reliability standards (Cronbach's alpha and intra-class correlation > 0.7), except for social relationships (alpha = 0.55). It is also reported that it discriminated well between subgroups of the study samples differing in their health status and demonstrated statistically significant correlation with the Iranian version of the SF-36 (14). Another study has supported the validity of the questionnaire among women in the postpartum period. All domains of the WHOQOL-BREF met reliability standards (alpha coefficient > 0.70) and the questionnaire discriminated well between depressed and non-depressed groups (P < 0.001) and showed satisfactory correlations with the Australian Unity Well-being index (r ≥ 0.45) (15). We examined the internal consistency of the questionnaire using the Alpha Cronbach coefficient.

#### 3.1.3. GHQ28

The General Health Questionnaire (GHQ) is one of the screening tools used in epidemiological studies of psychiatric disorders. The 28-item version of GHQ has been used for screening various groups including women in ante- and postnatal periods (16). The GHQ contains 28 questions in four domains: somatic symptoms, anxiety and insomnia, social dysfunction, and severe depression. Each domain has 7 items which have a 4 point scoring system from zero to 3 based on a 4-point Likert scale. A higher score implies a more unfavorable psychological status. We followed the standard procedure to score missing data in the GHQ, which counted omitted items as low scores. Previous studies have supported the validity and reliability of Farsi version of GHQ28 (17, 18). The cut-off point in Iran has been calculated at 24 (17).

#### 3.1.4. Breastfeeding Experience Scale

Breastfeeding Experience Scale (BES) is a questionnaire that consists of 30-items measuring breastfeeding practices, experiences, and outcomes. In this study, we used the first 18 questions, which rate severity of common breastfeeding difficulties in the early postpartum period using a 5‐point numerical rating scale (1 = not at all, 5 = severe). To score missing data in the BES, we counted omitted items as low scores. Responses are then summed up to obtain a total breastfeeding problem severity score (range 18‐90), with a higher score representing increased problem severity. Content validity and internal consistency of this scale (alpha coefficient 0.76) was supported during early development of the BES. The results of principle components factor analysis with the data revealed that the 18 items fell into a five-factor solution: mechanics, breast, insufficient milk, social, and process concerns, which altogether explained 60% of variance (19). Also in another study, the internal consistency of the questionnaire at 3 and 6 weeks postpartum were 0.79 and 0.72 respectively (20). For this study, the alpha coefficient was (0.82) at 4 weeks postpartum. At the first step, we got permission from Professor Wamback to use the instrument. Then the instrument was translated in Farsi and assessed in a panel of experts in obstetrics and pediatrics. Content validity assessment of the questionnaire was performed using the Content Validity Index (CVI). CVI of all 18 items of the instrument was 0.8 to 1. No items were changed. A PhD in English language then back translated it and compared with the original instrument. There was no discrepancy in items’ meaning. Then we tested the Farsi version of the questionnaire for readability and ease of administration prior to the study in a pilot study. In addition, we conducted Exploratory Factor Analysis (EFA) on 18 items. We instructed the software to extract all factors with eigenvalue higher than 1. We found that the Farsi version of the first 18 items of the BES included 5 factors, which explained 58.57% of variance.

#### 3.1.5. Statistical Analysis

Statistical analysis was performed using SPSS 18 (SPSS Inc., Chicago, IL, USA). All independent metric variables were not normally distributed. Paired t-test was utilized to examine the changes of QOL from ante- to postpartum period. We conducted linear regression analyses to find variables, which were significantly related to QOL. In addition, we used Scatter diagrams to visualize the relationship between dependent variable and each metric independent variable to check and be sure they are linear. In addition, we checked whether the relationship between dependent variable and dichotomous independent variables is homoscedastic. We also checked whether the distribution of errors were normal. In order to determine the factors that predict QOL, two multiple regressions analyses were conducted. Multicollinearity between independent variables was tested before the analyses. For the first analysis the tolerance was 0.942 to 1.00 (i.e., higher than the standard value of 0.1), and the variance inflation factor was 1.00 to 1.166 (i.e., lower than the standard value of 10). For the second analysis, tolerance was 0.964 to 1.00, and the variance inflation factor was 1.001 to 1.038 which indicated the absence of multicollinearity. The significance level of tests was set at 0.05.

## 4. Results

### 4.1. Women’s Characteristics

Mean age of women was 26.17 with ages ranging from 15 to 42 years. Median monthly income of the household was 4 million RLS. Forty-nine percent owned their homes. The educational level of women was primary school 11%, lower secondary school 17%, upper secondary school 44%, and university 28%. About 91.1% of the sample was homemaker, 41.3% were multigravida, 6% suffered from a mild to moderate chronic disease, 23% had a pregnancy complication and only two women stopped breastfeeding during the study period. The prevalence of cesarean and unwanted pregnancy in our sample was 53% and 12%, respectively. None of them smoked or had a history of smoking, but 7% reported that their husbands smoked. All were married.

### 4.2. Internal Consistency of WHOQOL-BREF

The values of Alpha Cronbach coefficient for each of the four subscales and the whole WHOQOL-BREF questionnaire in the ant- and postpartum periods were, respectively: physical subscale 0.83 and 0.82, psychological 0.78 and 0.73, social relationship 0.77 and 0.68, environmental 0.79 and 0.80 and the whole questionnaire 0.92 and 0.93.

### 4.3. Comparison of QOL in Ante- and Postnatal Periods

Table 1 shows means and standard deviations of QOL in ante- and postnatal periods. In antenatal and postnatal period, the lowest mean value of the scores belongs to the physical subscale and the psychological subscale respectively.

**Table 1.** Distribution of Means and Standard Deviations of QOL and Changes in QOL between Ante- and Postnatal Periods ^a^

	Pregnancy	Postpartum	Mean Difference		
				95% CI	P value
**Physical**	62.96 ± 17.2	70.88 ± 15.42	0.91 ± 17.23	6.07, 9.75	< 0.001 ^b^
**Psychological**	63.59 ± 16.63	64.15 ± 17.15	0.56 ± 15.21	**−**1.06, 2.19	0.494
**Social**	66.34 ± 20.54	68.63 ± 18.94	2.28 ± 19.68	0.18, 4.38	0.033^c^
**Environmental**	68.81± 14.81	67.92 ± 15.1	**−**0.88 ± 13.42	**−**2.31, 0.54	0.226
**Q1 ** ^**d**^	4.05 ± 0.71	4.03 ± 0.75	**−**0.01 ± 0.69	**−**0.09, 0.05	0.642
**Q2 ** ^**e**^	4.04 ± 0.81	3.93 ± 0.82	**−**0.1 ± 1	**−**0.21, 0.001	0.052
**Total**	66.32 ± 13.7	68.38 ± 13.6	2.05 ± 11.97	0.78, 3.32	0.002^f^

^a^ Abbreviations: CI, confidence interval.

^b^ P < 0.001.

^c^ P < 0.05.

^d^ Overall perceived QOL.

^e^ Overall perceived health.

^f^ P < 0.01.

In the antenatal period, 27% of women evaluated their overall QOL as very good, 54% as good, 18% as ‘not good, not bad’, 8% as bad and 0.3% as very bad. The corresponding figures for the postnatal period were 26.5%, 52.8%, 19%, 0.9% and 0.9%, respectively. In the antenatal period, 29% of women evaluated their overall health as very good, 52% as good, 16% as ‘not good not bad’, 1.7% as bad and 1.7% as very bad. The corresponding figures for the postnatal period were 22.4%, 56%, 15.5%, 5% and 1.2%, respectively.

In the antenatal period, the score of less than 50 in each of the physical, psychological, social relationship, and environmental domain were 11%, 22%, 21%, and 12% respectively. In the postnatal period, the score of less than 50 in each of the physical, psychological, social relationship, and environmental domain were 22%, 22.5%, 24%, and 10.4%, respectively.

Table 2 compares the results of the present study with previous studies. The scores in all domains of the QOL were different from the corresponding figures in Nikpour’s study (13) and scores of the social relationship and environmental domain of the QOL were different with those of Zubaran’s study (21).

**Table 2.** Distribution of Means and Standard Deviations of QOL in Different Studies ^a,b^

Domains	Nikpour (2011) (13) (n = 420)		Zubaran (2009) (21) (n = 101)		Mortazavi (2011) (n = 340)	
	Mean ± SD	95% CI	Mean ± SD	95% CI	Mean ± SD	95% CI
**Physical**	73 ± 11	72-74	71 ± 16	68.2–74	71 ± 15	69-73
**Psychological**	71 ± 15	69.5-72.5	63.3 ± 16	60-66.5	64 ± 17	62-66
**Social**	74 ± 16	72.5-75.5	67.2 ± 19	63.4-71	69 ± 19	67-71
**Environmental**	71 ± 14	69.6-72.36	60.6 ± 12.2	58–63	68 ± 15	66-70

^a^ Abbreviations: CI, confidence interval.

^b^ All three studies were done on women in the postpartum period using the WHOQOL-BREF with the scores converted to a 0-100 scale.

Table 3 shows the results of multiple regressions analysis (backward method) to assess the effects of predictors of global score of QOL in the ante- and postnatal period.

Variables were entered as predictors into the regression model if there were a statistically significant association with QOL (P < 0.05). The regression of QOL in the antenatal period on four variables (BMI, GHQ score, parity, occupation) was performed. Only GHQ score remained in the model and accounted for 22% of the variance. The regression of QOL in the postnatal period on 11 variables (weight gain, mode of delivery, breastfeeding difficulties, GHQ score, parity, occupation, age, preconception health, and housing) was performed. Weight gain, mode of delivery, breastfeeding difficulties, GHQ score, and parity remained in the model and accounted for 44.9% of the variance.

Furthermore, we found that unwanted pregnancy and rehospitalization in the postpartum were predictors of scores of psychological domain of QOL. Family income and age were predictors of scores of environmental domain, and maternal occupation was a predictor of scores of social domain of QOL.

**Table 3.** Predictors of Mother’s Global Scores of QOL in Ante- and Postnatal Period ^a^

	B	S.E	Beta	Significant	95% CI for B	
					Lower Bound	Upper Bound
Antepartum						
Constant	67.293	0.872		< 0.001 ^b^	65.574	69.011
GHQ score ≥ 24 ^c^	−10.530	1.350	−0.469	< 0.001 ^b^	−13.190	−7.870
**Postpartum**						
Constant	76.323	2.638		< 0.001 ^b^	71.117	81.529
Vaginal delivery^c^	2.548	1.251	0.116	0.043 ^d^	0.079	5.018
Weight gain ^e^	−0.157	0.068	−0.133	0.022 ^d^	−0.291	−0.023
Parity	−2.251	0.778	−0.168	0.004 ^f^	−3.786	−0.715
BF difficulties	−0.250	0.073	−0.210	0.001 ^f^	−0.394	−0.106
GHQ score ≥ 24 ^c^	−12.262	1.415	−0.524	<0.001 ^b^	−15.055	−9.468

^a^ Abbreviations: CI, confidence interval.

^b^ P < 0.001.

^c^ Variable’s code = 1.

^d^ P < 0.05.

^e^ Pregnancy weight gain.

^f^ P < 0.01.

## 5. Discussion

We investigated the QOL of women in the third trimester of pregnancy and 2 month postpartum, a difficult and challenging period due to major changes affecting all aspects of a woman’s life such as giving birth, challenges of breastfeeding and transient mood disorders. In the present study, we used the WHOQOL-BREF to investigate QOL in the ante- and postnatal periods as well as the significant factors associated with mothers’ ante- and postpartum QOL. The value of Alpha Cronbach coefficient calculated for the different subscales of the WHOQOL-BREF indicated its high level of internal consistency in both periods. Results showed that majority of women enjoyed a reasonable QOL in both periods, were satisfied of their health, and had a good evaluation of their QOL. These findings are in agreement with Brazilian study on postpartum women 2 month postpartum (21). Comparison of the scores in the two periods indicated that women’s physical health and social relationship had improved. Our findings are in agreement with previous studies with regard to improvement in physical health (5, 22) and the absence of improvement in mental health (5). A study that compare pregnant women and community controls reported that pregnant women in the late pregnancy had significantly lower levels of functioning with regard to bodily pain, physical functioning, social functioning, vitality, and functional limitations. Those differences were detected in the postpartum, too. Scores on social functioning and functional limitations decreased during the postpartum and women reported improved perceptions of their general health in the postpartum (6).

Comparison of the scores obtained by women in Shahroud in different domains of QOL with those of women in Amol in Nikpour’s study (13) indicates that Shahroudi women have lower QOL in all the domains which considering the similarities between the two cities, seems unreasonable. Both cities are located in the north of Iran and have healthy environmental conditions and low population. This is contrary to our expectations and requires further investigation. In Nikpour's study, depressed women (i.e. those having scores higher than 13 on the Edinburgh questionnaire) were excluded from the sample in antenatal period. This may partly explain the difference in results and it underlines the effects of the psychological domain on other domains of the QOL. Another useful comparison of our results would be with the findings of Zubaran (21) about breastfeeding mothers in southern Brazil, which indicates that Shahroudi women in the postnatal period are more satisfied with their environment and social relationships than Brazilian women. Zubaran’s study was carried out in Caxias do Sul, the second largest city in the state of Rio Grande do Sul, with a population of 428000 in 2008 (23). In comparison, Shahroud is a less populated city. With regard to different results in the social relationships domain, notice should be taken of the role of cultural differences between the two countries.

In both the ante- and postnatal periods, the most significant factor affected all domains and the global score of the QOL of women was GHQ score. Various studies have demonstrated the correlation between QOL and depressive symptoms and the negative impact of depression on QOL (24). A Chilean study demonstrated a statistically significant correlation between the average score on the Edinburgh Postnatal Depression Scale and the somatic and emotional SF-36 scores, with depressed women presenting poorer results on the SF-36 items that measure vitality, and role limitations (25). Similar results were reported by other studies that showed a significantly lower score on all SF-36 subscales among women experiencing postpartum depression (26, 27). Our results indicated that breastfeeding difficulties affected all domains of QOL negatively. Results of a study of 379 primiparas indicated that breastfeeding difficulties such as pain, cracked nipples, milk stasis or mastitis are associated with higher levels of psychological stress during first weeks postpartum (28). Another study reported that women with negative breastfeeding experiences were more likely to have depressive symptoms at 2 months postpartum (29).

We found that weight gain in pregnancy affected negatively QOL in postpartum. In previous studies, the relationship between obesity and poor QOL was found. Walfisch et al. reported that women with depressive symptoms had higher weights than non-depressed women and there was a strong association between body weight and depression in both pregnant and non-pregnant women (30). In another study on adolescents a statistically significant relationship between BMI and general and physical health was found. Self-reported health was significantly lower in overweight or obese adolescents (31).

In our study, cesarean reduced the global QOL. An Iranian study reported that the vaginal delivery group had a better QOL for vitality and mental health at 8 weeks and for physical functioning at 12 weeks postpartum. Also, comparing the findings within each group showed that the vaginal delivery group improved more on physical health related QOL than the caesarean group (32).

Also, our results showed that multiparity affected negatively QOL in postnatal period. While an Australian study demonstrated that primiparous mothers experienced greater limitations due to physical difficulties (33), an Iranian study found that primigravid women in pregnancy had higher mean score in most dimensions of sf-36 than multigravid (34).

We found that lower family income, unwanted pregnancy, employment and higher age are factors negatively related to certain domains of QOL. Previous studies on women’s QOL in reproductive age demonstrated that lower age, income satisfaction, lower number of pregnancies, and higher body mass index were related to different dimensions of the QOL (35, 36).

### 5.1. Conclusions

Our results indicate that the WHOQOL-BREF is a reliable instrument for use in research on health care programs for pregnant and postpartum women. Most of the women participating in the present study were satisfied with their health status and generally described their QOL as good. Comparison of the prenatal and postnatal periods indicates that women’s health in physical and social domains has improved. Factor that adversely affected the global score of the QOL in antenatal period was GHQ score > = 24. Factors that adversely affected the global score of the QOL in postnatal period were GHQ score > = 24, higher pregnancy weight gain, cesarean, multiparity and breastfeeding difficulties during 8 weeks postpartum.

The time period covered by the present study was from the third trimester of pregnancy to 8 weeks postpartum. We recommend that future studies examine the trajectory of changes in QOL from the first trimester to the end of pregnancy. In this study breastfeeding was the predominant method of infant feeding among the participants, so the question is posed whether or not the observed postpartum QOL improvement is the result of breastfeeding. Thus, we recommended that in future studies the QOL of breastfeeding and non-breastfeeding mothers be compared.

### 5.2. Limitations and Strengths

To evaluate the QOL, this study used the WHOQOL-BREF, an instrument that is not specifically designed for pregnant women but covers four domains. However, a previous study comparing The Mother-Generated Index (MGI) with the WHOQOL-BREF has reported a strong correlation between the two questionnaires (21). Our study enjoys good sample size of pregnant women attending Shahroud Health Centers. With regard to the fact that antenatal care coverage at least once and four times was reported 98% and 94% respectively in Iran in 2006-2010 (37), the study sample is representative and our result can be generalized to all Shahroudi pregnant women.
